# Automatic Analysis of Retinal Vascular Parameters for Detection of Diabetes in Indian Patients with No Retinopathy Sign

**DOI:** 10.1155/2016/8423289

**Published:** 2016-08-08

**Authors:** Behzad Aliahmad, Dinesh Kant Kumar, Rajeev Jain

**Affiliations:** ^1^School of Engineering, RMIT University, Melbourne, VIC 3000, Australia; ^2^Save Sight Centre, Delhi 110033, India

## Abstract

This study has investigated the association between retinal vascular parameters with type II diabetes in Indian population with no observable diabetic retinopathy. It has introduced two new retinal vascular parameters: total number of branching angles (TBA) and average acute branching angles (ABA) as potential biomarkers of diabetes in an explanatory model. A total number of 180 retinal images (two (left and right) × two (ODC and MC) × 45 subjects (13 diabetics and 32 nondiabetics)) were analysed. Stepwise linear regression analysis was performed to model the association between type II diabetes with the best subset of explanatory variables (predictors), consisting of retinal vascular parameters and patients' demographic information. *P* value of the estimated coefficients (*P* < 0.001) indicated that, at *α* level of 0.05, the newly introduced retinal vascular parameters, that is, TBA and ABA together with CRAE, mean tortuosity, SD of branching angle, and VB, are related to type II diabetes when there is no observable sign of retinopathy.

## 1. Introduction

According to the World Health Organisation (WHO), the current estimates are that more than 62 million individuals are affected by diabetes mellitus (DM), with 79.4 million cases expected by 2030 [1, 2]. The success of the treatments such as the one started in south India [3] is closely related to early detection and explicit risk factors of diabetes. This is a public health challenge and a number of diabetic patients (estimated one in two) will remain undiagnosed [3]. There are a number of people who are hesitant in taking the blood test till the disease has advanced and for such cases the treatment options for retinopathy or neuropathy become very limited. There is an urgent need for alternate options to identify diabetic patients.

There have been successes with population screening for diabetic retinopathy (DR) using digital photography of the retina [4, 5]. Studies have shown that changes to both retinal nonvascular and vascular parameters are associated with different stages of DR [6]. There is also evidence of foveal and macular thickening to be an indicator of minimal nonproliferative DR [7, 8]. Vascular parameters such as venule dilation and larger retinal arteriolar calibre [9, 10], changes to the vasculature shapes and arteriolar branching angle, increased tortuosity [11–13], and Fractal Dimension (FD) of the retinal vascular network [14–16] are associated with DR. These methods, however, require significant manual supervision and are not suitable for automatic analysis.

This study has identified the association between retinal vascular parameters that can be automatically obtained without manual supervision with type II diabetes and no diagnosed DR. This work provides an explanatory model of diabetes from retinal vascular parameter and knowledge about candidate clinical information, using retinal images with no sign of DR. RIVAS, a retinal imaging software that automatically measures a range of parameters, was used. The parameters that have already been reported in the literature were measured along with introduction of two new parameters: total number and average variability of the acute branching angles.

The study has developed statistical models using retinal vascular parameters, patients' demographic information, and clinical parameters as the explanatory variables. This is a follow-up to a recent similar work where only FD of Optic Disk Centred (ODC) retinal images was analysed [17]. A potential impact of this study is to allow for development of a predictive mode in the future to identify diabetic patients with no DR during a routine visit to an ophthalmologist or an optometrist.

## 2. Subjects and Methods

### 2.1. Database

Experiments were conducted using database collected in Department of the Retina, Save Sight Centre Hospital located in Delhi. Approval for this study was granted by the Human Research Ethics Committee (HREC) of the Royal Melbourne Institute of Technology (RMIT University), Melbourne, Australia, and also by the Institutional Review Board at Save Sight Centre Hospital in accordance with the declaration of Helsinki (1975, as revised in 2004). All participants were respondents to a request advertised in the “Save Sight Centre” in Delhi. The purpose and experimental procedure in plain language were given to the participants in written form and also explained verbally. Written and oral consent was obtained from the participants prior to data collection.

All the volunteers self-evaluated themselves to be “reasonably active” and none of them were pregnant. The participants were classified into two groups of type II diabetes (case) with no observable retinopathy and nondiabetic (control) patients. The diabetic cases were confirmed by the patients' physician based on either (i) their fasting or (ii) postprandial glucose plasma levels being greater than 126 mg and 200 mg/decilitre, respectively. None of the diabetic patients had any observable intraretinal haemorrhages or venous beading, hard exudates, and neovascularisation according to the classification levels by International Clinical Disease Severity Scale for DR [18]. This was confirmed by an ophthalmologist after examination of both eyes.

The participants' demographic information, including age, gender, weight (kg), height (m), systolic and diastolic blood pressure (mmHg), skin fold (mm), and cholesterol level (LDL and HDL), was recorded. The participants' age was limited to a narrow range of 40 to 73 years to remove confounding effect of the age factor on the analysis outcome and provide a better balance between the number of diabetic cases and the control groups. All the participants were nonsmoker, did not consume alcohol, had no history of any cardiovascular disease, and did not have any history of antihypertensive and lipid-lowering medications.

One ODC and one Macula Centred (MC) fundus images were taken from both eyes of each subject making total of four images for each participant. The photographs were taken in mydriatic mode in a dimmed light room using a mydriatic Kowa Vx alpha camera (Kowa, Japan). The original image resolution was 300 dpi (4288 × 2848 pixels) and the camera was set to an angle of 30°. All the images were examined in pairs for quality assurance (i.e., vessel to background contrast and illumination artefacts). After checking the quality discarding ungradable images and age adjustment, 180 retinal images (Two (left and right) × Two (ODC and MC) × 45 subjects (13 diabetics and 32 nondiabetics)) were obtained. The demographic information of the patients at the baseline has been provided in Table 1. In this study, ODC and MC images were analysed separately, but for each image category (i.e., ODC/MC) the retinal vascular parameters of the left and right eyes of each subject were averaged.

**Figure 1:**
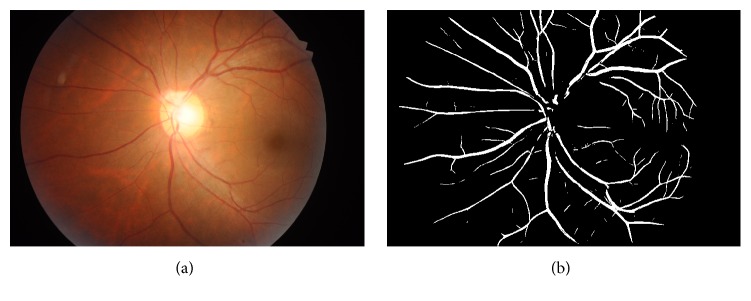
Example of ODC retina image of a diabetic patient: (a) RGB fundus image and (b) vessel-enhanced image in binary format (segmented image).

All the original images were first cropped and resampled to identical sizes of 729 × 485 pixels. Image enhancement and segmentation (binarization) was performed to reduce degrading image background artefacts and improve vessel to background contrast. This process was performed using mlvessel v1.3, software provided online by Soares et al. [19]. An example of the enhanced retinal vessels has been shown in Figure 1. For more information on the exact technique please refer to [20].

### 2.2. Retinal Vascular Parameter Measurement

Retinal vascular geometry features were measured quantitatively using unsupervised Retinal Image and Vasculature Assessment Software (RIVAS) v1.0 which has been specifically developed by the authors and described in [17]. In brief, the software combines the individual measurement into summary indices of multiple measurement options. Some of these are (i) vessel calibre of a specified segment, (ii) simple tortuosity, (iii) number of different Fractal Dimensions (FD), (iv) vessel-to-background ratio/percentage (V/B (%)),(v) average of acute branching angles (ABA) defined as the smallest angle between two daughter vessels, and (vi) the total number of branching angles (TBA). The FD measures include binary and grayscale Box-Counting (also known as Differential Box-Counting (DBC)) [21] and Fourier Fractal Dimension (FFD) [22].

**Figure 2:**
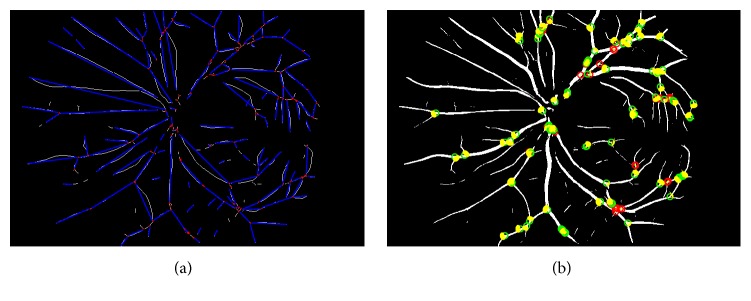
Example of branch point and vessel tortuosity detection: (a) vessel tortuosity measured using a thinned vascular network and (b) branch points and acute angle mapped on vessel-enhanced image in binary format (segmented image).

In this study, simple tortuosity was measured as the ratio between the actual length of a vessel segment and the shortest (Euclidean) distance between the two endpoints within the same segment providing a reflection of the shape/curvature of the vessel. Binary Box-Counting FD was calculated on skeletonized image as indicator of vascular network complexity without comprising any vessel calibre information. Example of some of these features has been shown in Figure 2.  Figure 2(a) shows the thinned vascular network (skeletonized) and the candidate points for location of the branches (red dots). The blue line in this figure is the shortest path connecting the two end points of the vessel segments identified by two consecutive true candidate branch points. Prior to segment identification, the candidate points were automatically labelled in RIVAS, as true (green circle) and false (red circle) branch points as in Figure 2(b). True candidates were defined as the points which correspond to the bifurcations (junction between two daughter vessels and a mother vessel) and false candidates were defined as the points corresponding to other possibilities: crossovers or noise.

Vessel diameter summary was also measured using IVAN software (University of Wisconsin, Madison, WI, USA) based on the calibre summary of the biggest 6 arterioles and venules separately and vessel summary represented by Central Retinal Arteriolar Equivalent (CRAE) and Central Retinal Venular Equivalent (CRVE) [23] as well as the ratio of the calibre of arterioles to venules (AVR). The measurements were performed within a fixed region and in between the margins of 0.5 to 1.0 disc diameters from the disc margin. CRAE and CRVE were obtained based on the revised Knudtson-Parr-Hubbard formula [23].

### 2.3. Statistical Analysis

Statistical analyses were performed using Minitab® v.16.1.0 and R studio (R® statistical software v.3.3.0). As the number of observations was relatively small, the data was statistically upsampled to the new size of 200 samples (i.e., 58 diabetic cases and 142 nondiabetic) using the bootstrapping technique with sample replacement. The upsampled data was then standardized and centred to decrease the multicolinearity between an interaction term and its corresponding main effects as well as making categorical parameters such as gender, comparable with continuous parameters.

Sixteen predictors were used in this analysis: vessel tortuosity (both mean and standard deviation (SD)), ABA, SD of angle, TBA, CRAE, CRVE, AVR, FD (Binary Box-Counting dimension of skeletonized images), and VB plus the patient's demographic information (i.e., gender, age, systolic and diastolic blood pressure, Body Mass Index (BMI), and skin fold).

Multiple linear regression model was built using all the 16 parameters as dependent variable and diabetes status with two possible categories (i.e., case and control) as independent variable. Analysis of variance (ANOVA) test was performed and* F* statistic was calculated to check the model fit. *R* ^2^ was also calculated to examine if the model is close to the regression line and obtain the percentage from the dependent variable's variation explained by the model. For each predictor in the model the coefficients and their significance level were calculated to identify potential nonsignificant variables and remove them from the model. The test for multicolinearity was performed by looking into the Variance Inflation Factor (VIF) for standard error of the regression coefficients. VIF greater than 5 was considered as presence of high multicolinearity between the predictors.

This paper also reports a stepwise regression approach, where the aim was to improve the exploratory stages of model building, but without compromising the physiological understanding of the predictors by using methods such as principal component analysis (PCA) for dimensionality reduction. This is essential for medical applications because the clinicians are keen to identify the relevant health parameters along with improved labelling of the data. For this purpose, stepwise regression analysis was performed to select the variables that are significantly important. In this process the most important variables are first selected with a forward searching algorithm followed by a backward elimination process to provide a reduced model with most suitable variables. This method adds and removes the predictors in each step until all the variables used in the model have* P *value ≤ *α* and the ones which are not used in the model have* P *value > *α* with *α* = 0.05.

For each predictor in the reduced model, VIF was calculated to test for the multicolinearity followed by testing the model for fitting performance using ANOVA and *R* ^2^ statistics.

## 3. Results

The demographic information (mean ± SD) of the patients at the baseline prior to bootstrapping the data has been summarized in Table 1. The BMI was calculated as weight (kg) divided by squared height (m^2^).

### 3.1. Explanatory Model for Diabetes

Data analysis was performed separately on both ODC and MC images to identify the relationship between potential predictors and diabetes factor. The first model was applied to ODC images. The result from linear regression analysis and ANOVA test for the first full model has been shown in Table 2.

In this model, the *R* ^2^ of 96.6% indicates 96.6% from the diagnosis variation is due to the model (or due to change in predictors) and only 3.4% is due to error or some unexplained factors. *P* values of <0.05 represent the fact that predictors play a significant role in the regression model. The ANOVA test also shows that the multiple linear regression model fits well to the data (*F* = 3094.49, *P* < 0.001). However, in this model, VIF is greater than 5 for almost all the predictors (except diastolic blood pressure) showing that there is problem concerning the predictors' colinearity.

A second model was built from the full model using stepwise procedure to reduce the number of parameters and the multicolinearity of the predictors. The result has been provided in Table 3.

For the second model, ANOVA test shows that the multiple linear regression model fits significantly with the data (*F* = 50.11, *P* < 0.001). The VIF factor in all cases is smaller than 5 showing negligible colinearity between predictors. In this model the coefficients are weaker than the first model with reduced *R* ^2^. This result is in line with general expectations that there is reduction in model goodness of fit with reduction in number of features; however, this does not represent decline in the explanatory power of the reduced model compared to the full model. Also interpretation of the coefficients in the second model with reduced number of variables and negligible degree of colinearity is more valid and accurate compared to the full model. Comparison between the two models shows that some coefficients have lower magnitude in the second model. Also some predictors (i.e., CRAE, mean tortuosity, VB, and age) change their sign from the full model to the reduced model, resulting in some degree of uncertainty for the interpretation of the full model with highly colinear predictors.

The results also show that the retinal vasculature parameters with *α* level of 0.05 that play a significant role in the reduced explanatory model of diabetes are the CRAE, mean tortuosity, ABA, SD of branch angles, VB, and TBA. From the clinical and demographical information, only systolic blood pressure and age were found to be significant predictor.

The same analysis as explained above was performed on ODC images; however, no model was found to provide adequate fit to the MC data for this database.

## 4. Discussion

This research has studied retinal vasculature parameters to detect DM in the Indian population with no DR. The analyses were performed on both MC and ODC images using a 30° nonmydriatic eye-fundus camera. The significance of this work is that it reports automatic analysis of the eye-fundus images and provides an explanatory model for early changes in some retinal vascular parameters as a result of DM. This study has also introduced a set of new retinal vascular parameters, TBA and ABA, and employed them together with other features and patients demographic information to create an explanatory model for prediction of diabetes in the absence of evident features of diabetic retinopathy.

In this work, linear regression analysis was performed to model association between a large number of explanatory variables as the predictors (i.e., retinal vascular parameters and clinical information) and DM as the response variable. The application of stepwise regression allowed for dimensionality reduction as well as solving the multicolinearity problem making the send model clinically interpretable. It was important that predictors should be clinically relevant and without compromising the physiological understanding of the predictors. The result showed that six retinal vascular parameters of (1) CRAE, (2) mean tortuosity, (3) ABA, (4) SD of angle, (5) VB, and (6) TBA are associated with diabetes in Indian population when there is no observable DR. The ANOVA test and resulting adjusted *R* ^2^ indicated a good fit of the model to the data.

The significant outcome of this study is that it provides the basis for an alternate technique to detect diabetes among people with minimal or no DR. This could be very useful for people who are hesitant in taking blood tests.

Another major outcome of this study is that it has introduced two retinal vascular parameters that may find application in a predictive model to predict the risk of developing DR in diabetic patients with no DR. Therefore, conducting longitudinal study is proposed to monitor the progress of the patients and identify changes among those who may later develop DR. The measurements are suitable for automation and for being used with simple eye-fundus imaging, which could be put in form of a modified smart-phone in the future [24].

The limitation of this study is that it was limited in the population type, having been conducted only in Delhi in India, and represents narrow demographics with limited number of samples and matching cases. This may lead to limited diagnostic utility for the current model. It is essential to include subjects with similar cultural, ethnic, and socioeconomic conditions. There is also the need for conducting similar tests on a larger dataset and for other demographics to observe potential differences and better evaluate the performance of the model.

## Figures and Tables

**Table 1:** Baseline characteristics of diabetic and nondiabetic subjects (metadata) at the baseline prior to bootstrapping.

Patients' characteristics	Diabetic (*n* = 13)			Nondiabetic (*n* = 32)		
	Mean ± SD	Min	Max	Mean ± SD	Min	Max
Age (years)	56 ± 7.25	46	67	51.93 ± 9.00	40	73
Systolic blood pressure (mmHg)	143.84 ± 21.90	110	185	131.55 ± 12.53	100	150
Diastolic blood pressure (mmHg)	81.15 ± 11.20	60	100	80.34 ± 11.48	55	110
Weight (kg)	75 ± 15.45	51	105	71.58 ± 18.04	35	115
Height (m)	1.65 ± 0.09	1.50	1.82	1.64 ± 0.09	1.46	1.82
BMI (Kg/m^2^)	27.34 ± 3.97	17.64	32.71	26.52 ± 6.42	14.02	41.91
Skin fold (mm)	38.69 ± 4.11	33	46	36.86 ± 6.02	24	51
Gender (female/male)	7/6			12/20		

**Table 2:** Predictors coefficients, significance level for the first model, and ANOVA test result.

Predictor	Coef	SE	*T*	*P*	VIF	Adjusted *R* ^2^	ANOVA	
							*F*	*P*
Constant	0.46335	0.01242	37.31	<0.001		96.6%	3094.49	<0.001
CRAE	11.9878	0.0931	128.81	<0.001	1493.583			
CRVE	−4.20147	0.03378	−124.39	<0.001	238.956			
AVR	−11.7099	0.0905	−129.43	<0.001	1631.594			
Mean tortuosity	−0.5174	0.01389	−37.26	<0.001	55.644			
SD of tortuosity	0.150746	0.008002	18.84	<0.001	18.545			
ABA	2.54491	0.02117	120.2	<0.001	49.765			
SD of angle	1.68918	0.01853	91.14	<0.001	36.814			
VB	0.82474	0.02233	36.93	<0.001	155.256			
TBA	0.960501	0.009531	100.77	<0.001	22.108			
FD	−0.81633	0.01878	−43.47	<0.001	94.546			
Gender	1.1179	0.0097	115.27	<0.001	18.8			
Age	−0.53138	0.01118	−47.52	<0.001	12.804			
BMI	0.02697	0.011	2.45	0.015	13.547			
Systolic blood pressure	0.441949	0.004433	99.71	<0.001	5.542			
Diastolic blood pressure	−0.22795	0.005911	−38.56	<0.001	4.476			
Skin fold	−0.32616	0.01465	−22.27	<0.001	19.118			

**Table 3:** Predictors coefficients, significance level for the second model, and ANOVA test result.

Predictor	Coef	SE	*T*	*P*	VIF	Adjusted *R* ^2^	ANOVA	
							*F*	*P*
Constant	0.312	0.015	20.11	<0.001		44.95%	50.11	<0.001
CRAE	−0.088	0.018	−4.71	<0.001	1.47			
Mean tortuosity	0.219	0.027	8.13	<0.001	3.01			
ABA	0.239	0.038	6.24	<0.001	4.1			
SD of angle	0.179	0.040	4.45	<0.001	4.78			
VB	−0.269	0.030	−8.88	<0.001	3.82			
TBA	0.262	0.022	11.48	<0.001	2.17			
Age	0.111	0.020	5.44	<0.001	1.73			
Systolic blood pressure	0.108	0.017	6.11	<0.001	1.31			
